# Family physicians overestimate diagnosis probabilities regardless of the test results

**DOI:** 10.3389/fmed.2023.1123689

**Published:** 2024-01-08

**Authors:** Ömer Ataç, Hüseyin Küçükali, Ayşe Zülal Tokaç Farımaz, Ayşe Seval Palteki, Sabanur Çavdar, Melek Nur Aslan, Muhammed Atak, Mehmet Akif Sezerol, Yusuf Taşçı, Osman Hayran

**Affiliations:** ^1^Department of Public Health, International School of Medicine, Istanbul Medipol University, Istanbul, Türkiye; ^2^Department of Health Management and Policy, College of Public Health, University of Kentucky, Lexington, KY, United States; ^3^Department of Public Health, School of Medicine, Istanbul Medipol University, Istanbul, Türkiye; ^4^Centre for Public Health, Queen’s University Belfast, Belfast, United Kingdom; ^5^2022-2023 Hubert H. Humphrey Fellow, Rollins School of Public Health, Emory University, Atlanta, GA, United States; ^6^Fatih District Health Directorate, Istanbul, Türkiye; ^7^Department of Public Health, Hamidiye Institute of Health Sciences, University of Health Sciences, Istanbul, Türkiye; ^8^Department of Public Health, Istanbul Faculty of Medicine, Istanbul University, Istanbul, Türkiye; ^9^Department of Epidemiology, Graduate School of Health Sciences, Istanbul Medipol University, Istanbul, Türkiye; ^10^Sultanbeyli District Health Directorate, Istanbul, Türkiye; ^11^Üsküdar District Health Directorate, Istanbul, Türkiye

**Keywords:** primary care, family medicine, general practice, diagnostic tests, likelihood ratios

## Abstract

**Introduction:**

As useful tools for clinical decision-making, diagnostic tests require careful interpretation in order to prevent underdiagnosis, overdiagnosis or misdiagnosis. The aim of this study was to explore primary care practitioners’ understanding and interpretation of the probability of disease before and after test results for six common clinical scenarios.

**Methods:**

This cross-sectional study was conducted with 414 family physicians who were working at primary care in Istanbul via face-to-face interviews held between November 2021 and March 2022. The participants were asked to estimate the probability of diagnosis in six clinical scenarios provided to them. Clinical scenarios were about three cancer screening cases (breast, cervical and colorectal), and three infectious disease cases (pneumonia, urinary tract infection, and COVID-19). For each scenario participants estimated the probability of the diagnosis before application of a diagnostic test, after a positive test result, and after a negative test result. Their estimates were compared with the true answers derived from relevant guidelines.

**Results:**

For all scenarios, physicians’ estimates were significantly higher than the scientific evidence range. The minimum overestimation was positive test result for COVID-19 and maximum was pre-test case for cervical cancer. In the hypothetical control question for prevalence and test accuracy, physicians estimated disease probability as 95.0% for a positive test result and 5.0% for a negative test result while the correct answers were 2.0 and 0%, respectively (*p* < 0.001).

**Discussion:**

Comparing the scientific evidence, overestimation in all diagnostic scenarios, regardless of if the disease is an acute infection or a cancer, may indicate that the probabilistic approach is not conducted by the family physicians. To prevent inaccurate interpretation of the tests that may lead to incorrect or unnecessary treatments with adverse consequences, evidence-based decision-making capacity must be strengthened.

## 1 Introduction

Diagnostic tests are helpful tools to facilitate deciding the correct diagnosis in line with the medical history and symptoms of the patients in terms of clinical decision-making (1). Application of the principles of evidence-based medicine helps clinicians make better diagnostic and management decisions. All diagnostic procedures, including laboratory tests, are based on probability estimations that need careful interpretation. Unnecessary request or misinterpretation of diagnostic tests may lead to underdiagnosis, overdiagnosis or misdiagnosis (2). Misinterpretation can adversely affect treatment, recovery, and health expenditures (3).

The accuracy of the tests which can be estimated as sensitivity, specificity and predictive values is decisive when deciding on diagnosis and treatment. The prevalence of diseases is also a measure which strongly influences the positive predictive value: the lower the prevalence, the lower is the probability of being sick even after a positive test (4).

Methods regarding accuracy, reproducibility and probability estimations for diagnostic tests are provided to medical school students through clinical epidemiology and evidence-based medicine curriculum (5). However, studies show that physicians do not comprehensively understand and interpret the probabilities during their clinical practices (6). The main consequences of this phenomena is overestimation of both positive and negative test results by physicians (7–9). The same problem exists among primary care physicians who have a key role in screening programs and outpatient services (10).

In the Turkish healthcare system primary care and preventive services are provided by family physicians for the registered populations (11). Family physicians are general practitioners (GP) or specialists and have an important role in the screening in breast, cervical and colorectal cancers as a part of national control programs in addition to primary care practices (12). Respiratory system and urinary tract infections are among the most common causes of admission to their offices (13). Therefore, family physicians are expected to use and interpret the test results appropriately and estimate correct probabilities of these diseases. This issue has become more important during the COVID-19 pandemic when tests for infection detection were widely performed, and their correct interpretation was important (14).

In this study, our aim was to explore primary care practitioners’ understanding and interpretation of the probability of a disease before and after test results for six common clinical scenarios.

## 2 Materials and methods

This cross-sectional study was conducted among family physicians working at primary care services in Istanbul during November 2021 and March 2022. Sample size was calculated as 354 assuming a prevalence of risk overestimation of 50%, with 95% confidence intervals within ±5%. All primary care physicians in five geographically dispersed districts of İstanbul (Başakşehir, Eyüpsultan, Fatih, Sultanbeyli, Üsküdar) were included in the study without using any sampling method (*n* = 613). Among them, 414 physicians have participated in the study, with a 67.5% response rate. Data was collected during face-to-face interviews.

The questionnaire consisted of two sections. The first section contained seven questions regarding the participants’ sociodemographic and professional characteristics. The second section contained questions about the probability of diseases in six clinical scenarios (Appendix 1). These scenarios are adapted from the study of Morgan et al. (10) in consideration of the common health problems which are expected to be diagnosed and/or treated by family physicians in Türkiye. Scenarios included three cancer types (breast, cervical and colorectal) within scope of the national cancer control program, two frequent infectious diseases (pneumonia and urinary tract infection) in primary care, and ongoing COVID-19 pandemic. Cases in the cancer screening scenarios were asymptomatic while they were symptomatic in infectious disease scenarios. Each scenario was prepared in agreement with the recent literature. A hypothetical control question measuring the understanding of the participants on the accuracy of diagnostic tests was also included.

Mammography for breast cancer, pap smear for cervical cancer, stool occult blood test for colorectal cancer, chest radiography for pneumonia, complete urinalysis for urinary tract infection, and PCR test for COVID-19 were used as diagnostic tests and the participants were asked probability of the given disease in three conditions: (a) before performing the diagnostic test, (b) after a positive test result, and (c) after a negative test result. The participants’ responses were compared to the test accuracy values from existing evidence-based literature. True answers to the questions were determined considering the most relevant national and international guidelines for physicians in Türkiye (Appendix 1).

Python programming language was used for data analysis. Descriptive statistics to summarize the data were frequency, percentage, median, interquartile range (IQR) for non-normally distributed continuous variables, the mean, and the standard deviation for normally distributed continuous variables. The Wilcoxon signed-rank test was used to compare physicians’ estimates of disease probabilities and probabilities derived from the evidence. Type I error (α) level of 0.05 was used in the interpretation of the significant test results.

This study was conducted with the permission from the Ministry of Health of the Turkish Republic (07/10/2021) and was approved by the Ethics Committee of Non-Invasive Clinical Studies of Istanbul Medipol University (15/10/2021, No: 1021).

## 3 Results

414 family physicians participated in the study. 53.9% (*n* = 223) of them were male, 86.2% (*n* = 357) general practitioners and the mean age was 42.9 ± 10.3. The characteristics of the study sample are summarized in Table 1.

**Table 1:** Descriptive characteristics of the participants.

		*n*	%
District	Üsküdar	104	25.1
	Fatih	101	24.4
	Eyüp	74	17.9
	Başakşehir	74	17.9
	Sultanbeyli	61	14.7
Gender	Male	223	53.9
	Female	187	45.2
	Unknown	4	1.0
Physician group	General practitioner	363	87.7
	Specialist	44	10.6
	Unknown	7	1.7
Total		414	100.0
		*n*	Mean ± S.D.
Age		410	42.9 ± 10.3
Professional experience (years)		404	16.9 ± 10.5
Experience as GP (years)		370	7.6 ± 4.0
Number of registered populations		405	3373.5 ± 860.2

**Figure 1:**
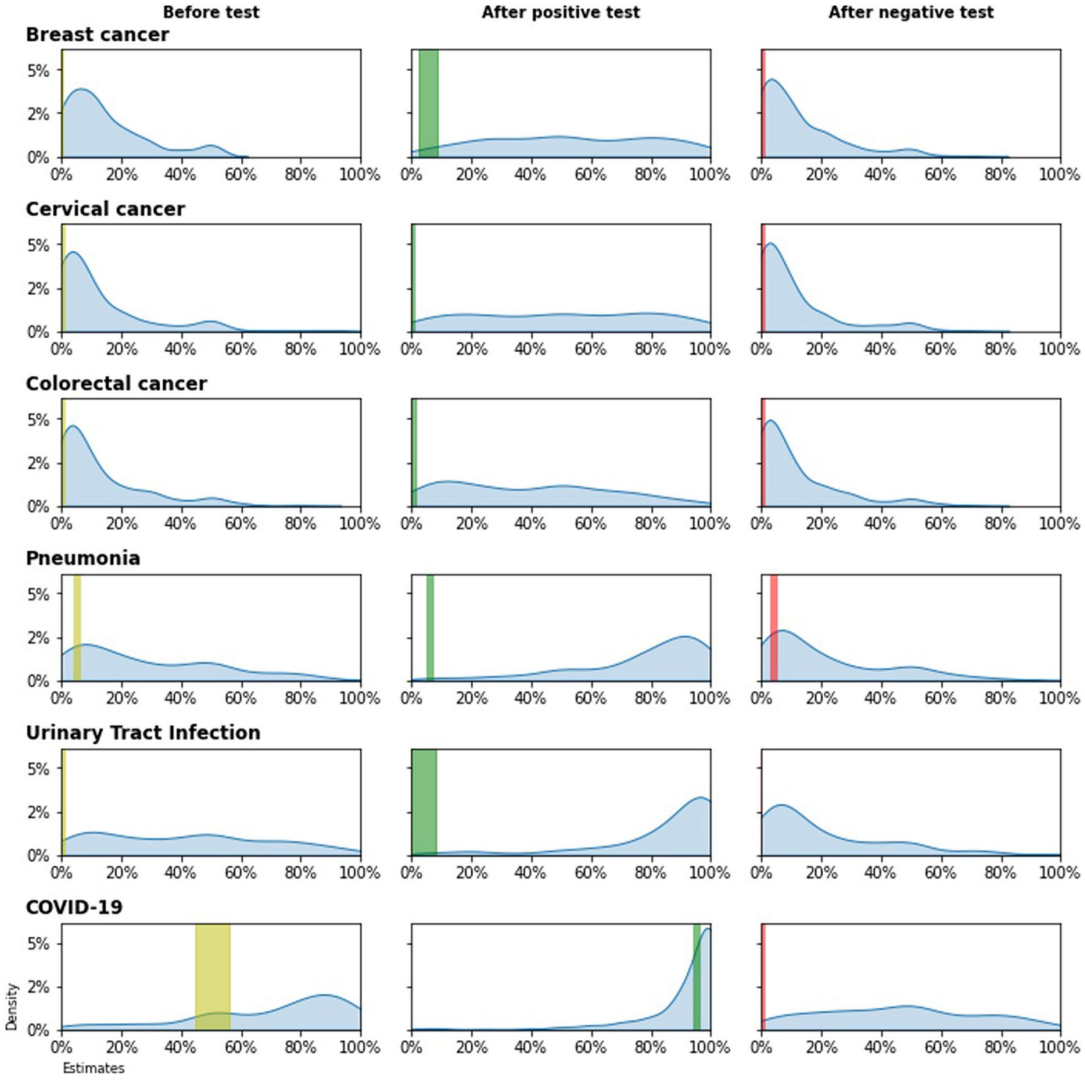
Estimates of diagnostic probabilities for each case description* *Colored vertical bars indicate the evidence range.

The density distribution of physicians’ estimates of diagnostic probabilities for each scenario is presented in Figure 1. For all scenarios, physicians’ estimates were significantly higher than the scientific evidence range (Appendix 2; Supplementary Table S1).

For breast cancer, the median of physicians’ estimation was 10.0% (evidence was 0.2%) for pre-test disease probability (*p* < 0.001). Both for cervical and colorectal cancer, the median of physicians’ estimation was 5.0% (evidence was, respectively, 0.01 and 0.06%) for pre-test disease probability (for both comparisons, *p* < 0.001). After a positive test result, physicians estimated the disease probability as 50.0% both for breast cancer and cervical cancer while evidence was, respectively, 8.7 and 0.14% (for both comparisons, *p* < 0.001). After a positive test result, physicians estimated the colorectal cancer probability as 40.0% (evidence was 0.74%; *p* < 0.001). When test results were negative, physicians estimated the disease probability as 10.0% (evidence was 0%) for breast cancer, as 5.0% (evidence was 0.0032%) for cervical cancer and 5.0% (evidence was 0.02%) for colorectal cancer (for both comparisons, *p* < 0.001).

For pneumonia and urinary tract infection, physicians’ estimations were 20.0% and 40.0%, respectively, for pre-test disease probability, while the evidence was 5.0% and 1.0%. After a positive test result, these estimations increased to 85.0% and 90.0% respectively, while the evidence was less than 10.0%. When the test results were negative, physicians estimated the disease probability as 10.0% whereas the evidence was less than 5.0%.

For COVID-19, the median of physicians’ estimation was 80.0% (IQR 50.0–90.0%) for pre-test disease probability while evidence was 56.0% (*p* < 0.001). After a positive test result, physicians estimated the disease probability as 99.0% (IQR 90.0–100.0%) whereas evidence was 95.4% (*p* < 0.015). When test results were negative, physicians estimated the disease probability as 50.0% (IQR 25.0–70.0%) while evidence was 0.04% (*p* < 0.001).

When physicians were provided with prevalence and test accuracy information of the hypothetical control question, they estimated disease probability as 95.0% (IQR 95.0–100.0%) for a positive test while the true answer was 2.0% (*p* < 0.001) and 5.0% (IQR 5.0–10.0%) for a negative test whereas the true answer was 0% (*p* < 0.001).

Table 2 shows the breakdown of disease probability estimations by physician groups. Although specialists showed a slightly lower tendency toward overestimation than general practitioners in several scenarios, their estimates were not close to the evidence ranges. Subgroup analyses showed that female participants estimated probabilities higher than males with a median difference around the range of 2.0–15.0, but estimations of participants were consistent across districts (Appendix 2; Supplementary Tables S2–S3).

**Table 2:** Estimated disease probabilities for each scenario by physician groups.

Scenarios	Physician Groups, Median (IQR)			Evidence range*, %
	GPs, %	Specialists, %	Both groups, %	
Breast cancer				
Before test	10 (5–20)	10 (4–16)	10 (5–20)	0.2–0.3
After positive test result	50 (30–80)	50 (30–80)	50 (30–80)	2.5–8.7
After negative test result	10 (2–20)	8 (1–20)	10 (2–20)	0
Cervical cancer				
Before test	6 (2–20)	4 (1–10)	5 (2–20)	0.01
After positive test result	50 (22–80)	50 (20–72)	50 (20–80)	0.14
After negative test result	5 (1–15)	3 (1–10)	5 (1–13)	0.0032
Colorectal cancer				
Before test	7 (2–20)	4 (2–10)	5 (2–20)	0.06
After positive test result	40 (15–60)	25 (10–50)	40 (15–60)	0.74
After negative test result	5 (1–15)	4 (1–10)	5 (1–15)	0.02
Pneumonia				
Before test	20 (10–50)	20 (10–50)	20 (10–50)	5
After positive test result	85 (65–90)	80 (75–90)	85 (70–90)	6.2
After negative test result	10 (5–30)	20 (9–38)	10 (5–30)	4.1
UTI				
Before test	45 (20–70)	20 (10–50)	40 (15–65)	0–1
After positive test result	90 (80–100)	90 (75–100)	90 (80–100)	0–8.3
After negative test result	10 (5–35)	10 (2–28)	10 (5–30)	0–0.11
COVID-19				
Before test	80 (50–90)	80 (55–88)	80 (50–90)	45–56
After positive test result	99 (90–100)	96 (90–100)	99 (90–100)	95.41
After negative test result	50 (25–70)	50 (30–68)	50 (25–70)	0.04
Control question				
After positive test result	95 (90–100)	95 (95–100)	95 (95–100)	2
After negative test result	5 (5–10)	5 (5–5)	5 (5–10)	0

UTI, Urinary Tract Infections; IQR, Interquartile range. *See Appendix 1 for the references to the evidence range derived through review of the literature.

Physicians’ probability estimations were significantly different from the evidence values (Appendix 2; Supplementary Table S4). Further subgroup analyses by physician group, gender, and district were provided in Appendix 2; Supplementary Tables S5–S7.

## 4 Discussion

In this study, we investigated diagnostic probability estimations of the family physicians about different scenarios regarding six hypothetical clinical cases. Three of the cases were pneumonia, COVID-19, and UTI, which are frequently encountered infectious diseases in primary care; the other three were cervical cancer, breast cancer, and colorectal cancer, which are routinely screened in primary care within the scope of the national cancer control program. Most striking finding of the study was the overestimation of all diagnoses before and after test results for the given scenarios.

The overestimation in all scenarios shows that the underlying potential causes of overestimation are not specific to the clinical cases but represents a more general problem. It is noteworthy that the overestimation varies between 1.04 times (COVID-19, after positive test) and 1,250 times (cervical cancer, before test), and it is over 10 times in 13 of the 18 alternatives examined. As was found in Morgan et al.’s study, these results are related to the overestimates of pre-test probability (10).

Overestimated responses given to the negative test results in all cases show that physicians overemphasize the symptoms when deciding on the diagnosis. Besides, the overestimated answers given to the hypothetical control question indicate that the physicians do not have comprehensive knowledge of probability estimations. The fact that the overestimation in our study was similar to previous studies reveals the universality of the problem. In a review article investigating how healthcare professionals interpret the results of diagnostic tests, it was stated that the probability estimates were in the direction of overestimating, regardless of whether the test result being positive or negative, and it has been concluded that commonly used measures of test accuracy are poorly understood by health professionals (7).

Family physicians in primary care have a key role in the management of clinical cases examined in our study. They work as individual health consultants who deal with all the health problems of their enrollees, provide preventive services and who are expected to solve the handleable problems at the primary care level, or to refer the complicated, unresolved cases to further levels and then follow up (15). Therefore, their role is important not only in curative services but also in primary and secondary prevention such as cancer screening.

In our study, pretest probability was higher in cancer screening tests than in UTI and pneumonia, whereas it was higher in UTI and pneumonia than in breast cancer screening in Morgan et al.’s study. High levels of overestimation in presented cancer scenarios can be concluded as the general perception of physicians toward cancer screening tests. Comparable results in other studies on this matter have also shown that physicians tend to overestimate the risk of cancer (16, 17).

Probability of UTI was more overestimated than pneumonia in before and after negative test scenarios and this result may be regarded because of physicians’ prioritization of the patient’s symptoms compared to the test results while diagnosing UTI. According to the literature for UTI management, pretest probability, which is estimated with the patient’s current symptoms, can significantly affect the post-test probability and in clinically high-risk cases, the post-test probability is evaluated as high even if the test result is negative (18).

There was also an overestimation in COVID-19 case scenarios. However, the frequency of overestimated responses given before test and after positive test result (1.43 and 1.04, respectively) were lower than all other case scenarios. We conclude this finding as the result of availability of up-dated information for COVID-19 management prepared by the Ministry of Health (19).

Diagnostic tests are valuable tools when evaluated together with the patient’s symptoms and lead the physician to an exact diagnosis. Although some of them have successful diagnostic performance, some are not the gold standard and only used for screening which requires advanced procedures to confirm diagnosis (17). In fact, diagnosis ultimately depends on the physician’s decision. Symptoms, test results and consultations are important parts to decide an appropriate diagnosis and treatment. Current developments in medicine provide test alternatives to physicians, but it is criticized as the dependency on technological solutions puts the experiences of physicians on the shelf and they mostly rely on the test results rather than their own experience (20). From this perspective, while diagnostic tests have a noteworthy role, they may cause overestimation, as seen in our study.

Physicians’ decision-making is considered critical for patient safety, as diagnostic errors and inappropriate treatments can harm patients, fail to address actual problems and waste resources (21, 22). It was shown that if family physicians misdiagnose, they mostly misregulate the treatment (23). Misinterpreting test results during decision-making can have adverse effects on the patients. For instance, in cases such as pneumonia and UTI that may require antibiotic treatment, the overestimation can lead to unfavorable outcomes in terms of antibiotic resistance (24).

We found another important overestimation in cancer screening tests. These tests are not definitive diagnostic tools, and they only lead to further procedures to confirm diagnosis. In cancer screenings, it can be interpreted as favorable that physicians attribute more value to screening tests in order not to miss probable cases. However, the risk of labeling for false positive individuals and its social and psychological consequences should always be kept in mind (25).

Post-test probability is expected to be estimated by considering pre-test probability (i.e., prevalence) and test accuracy (i.e., sensitivity and specificity). The reasons for physicians’ poor performance in probability estimation are due to lack of knowledge or misunderstanding (26). They mostly do not use likelihood ratio and pre-test probability in their estimations, eventually, test results have been overestimated (7, 27). In a study evaluating physicians’ probabilistic approach to the test results, no significant difference was found according to the type of data (sensitivity-specificity vs. likelihood ratios) (28). In another study investigating the change between the pre-test and post-test estimations according to the type of data shared, the successful estimations were 8% for sensitivity-specificity, 34% for the likelihood ratio, and 73% for the graphic form. Researchers have emphasized that clinicians may have difficulties understanding values that require arithmetic calculation, but it is easier for them to understand with visual tools such as graphs (29). Although we did not inquire about the reasons in our study, the poor performance in the hypothetical question may have been caused by a misunderstanding of the question due to the technical terms. An accurate estimation of the probability of the test results is a fundamental competence during clinical decision-making (30), and the lack of knowledge on methodological topics can be eliminated with evidence-based medicine training (31).

When the sensitivity and specificity are constant, the predictive values change according to the prevalence while evaluating the test results (32). However, most physicians cannot consider the prevalence when estimating the probability of disease after a positive test result, regardless of clinical experience and the institution they work for (26, 29). On the other hand, it is necessary to know the prevalence of the disease in a country or region in order to estimate probabilities correctly during a clinical decision, and ignorance of the frequency is considered a bias (20). Yet, the updated prevalence of major diseases usually is not available for the participants of our study. They should be shared and updated regularly by the health authorities.

Our study has some limitations. Scenarios prepared in line with the current scientific guidelines were directed to physicians. However, we do not know the real situation as cases applying in real world experiences may not fit these ideal scenarios. A limitation is that the diagnostic criteria change in time and there is not any guide specific to the population in which the study was conducted. Except for the COVID-19, international data could be used for probability estimations in scenarios instead of national ones. Because the data were collected as face-to-face observation, the participants may have stated differently from the Hawthorne effect compared to their daily practice. Despite these limitations, the overestimation was excessive in all clinical cases, and cannot only be explained by confounding variables or misinterpretation of the questionnaire.

In conclusion, in the present study, primary care physicians consistently overestimated the actual risk of disease regardless of the results of diagnostic or screening tests, despite the relatively high frequency of such diagnoses, and the availability of well performing tests with their performance parameters. This problem makes it difficult to fulfill patients’ needs as it reduces the accurate decision-making by physicians when selecting diagnosis and treatment. The findings of this study indicate the extensiveness and the magnitude of the problem and warrant interventions to improve the quality of primary care. However, to determine the kind of intervention that addresses the issue best, there is a need for qualitative studies to illuminate why and how physicians overestimate the disease probabilities.

## Data availability statement

The raw data supporting the conclusions of this article will be made available by the authors, without undue reservation.

## Ethics statement

This study involving humans were approved by Ethics Committee of Non-Invasive Clinical Studies of Istanbul Medipol University (15/10/2021, No: 1021). This study was conducted in accordance with the local legislation and institutional requirements. The participants provided their written informed consent to participate in this study.

## Author contributions

ÖA, HK, AF, AP, SÇ, MNA, MA, MS, YT, and OH: conception and design. MNA, MA, MS, and YT: acquisition of data. ÖA and HK: analysis. ÖA, HK, AF, AP, and SÇ: interpretation. ÖA, HK, AF, AP, SÇ, and OH: draft and revision.
