# Supplementary material for: Family physicians overestimate diagnosis probabilities regardless of the test results
Source: Front Med (Lausanne). 2024 Jan 8;10:1123689. doi: 10.3389/fmed.2023.1123689 (PMC10801057; doi:10.3389/fmed.2023.1123689)
Supplement: Supplementary file 1 [file Data_Sheet_1.PDF]

Supplementary Material

1 Appendix 1. Scenarios with answers

Breast Cancer

Ms. Ayşe, 45-years-old woman who applied to you to get information about breast cancer screening. She has no specific risk factors or symptoms for breast cancer.

a. How likely is Ms. Ayşe to have breast cancer based on this information? _______%

b. Ms. Ayşe's mammogram is positive. How likely is she to have breast cancer? _______%

c. Ms. Ayşe's mammogram is negative. How likely is she to have breast cancer? _______%

Answers

a) Pretest probability = **~0.2-0.3%**

b) Posterior probability if test positive = **2.5-8.7%***

c) Posterior probability if test negative = **~0%**

Evidence summary:

According to the most recent ACS breast cancer statistics, the 10-year probability of developing invasive breast cancer is 1.5% for women aged 40-50[1]. If we divide by year, we get 1.5/10 = 0.15% per year, 0.15% of ACS <1%.

In addition, according to the Cancer Statistics Yearbook, the age-standardized breast cancer rate in 2017 is 47.7/100.000[2].

Banks et al.: women aged 50-64 (higher risk age) found 0.59% pretest probability for each mammogram[3].

Barlow et al.: women from BCS consortium, 0.3% pretest probability[4].

Pretest probability = ~0.2-0.3%

Sensitivity = 77-95%; specificity= 94-97%[5].

Positive likelihood ratio= 77/6 and 95/3 = 12.8-31.7

Negative likelihood ratio = 23/94 and 5/97= 0.05-0.24

Posterior probability if test positive = 2.5-8.7%*

Posterior probability if test negative = ~0%

* The calculation was based on 77% sensitivity and 94% specificity at 0.002 prevalence, 95% sensitivity and 97% specificity at 0.003 prevalence.

Cervical Cancer

Ms. Fatma, a 40-year-old woman, applied to you to get information about cervical cancer screening. She has no specific risk factors or symptoms for cervical cancer.

a. How likely is Ms. Fatma to have cervical cancer based on this information? _______%

b. Ms. Fatma's Pap-smear test is positive. How likely is she to have cervical cancer? _______%

c. Ms. Fatma's Pap-smear test is negative. How likely is she to have cervical cancer? _______%

Answers

a) Pretest probability = **0.01%**

b) Posterior probability if test positive = **0.14%**

c*) Posterior probability if test negative* = **0.0032%**

Evidence summary:

The prevalence of cervical cancer between the ages 40 and 44 was reported as 7.1 per 100,000 in 2020[6].

Pretest probability = 0.01%

In a systematic review conducted in 2016, the values of Pap smear test compared to HPV test, were determined as 70% sensitivity and 95% specificity[7].

Positive likelihood ratio = 70/(1-95) = 14.0

Negative likelihood ratio = (1-70)/95 = 31.6

Posterior probability if test positive = 0.14%

Posterior probability if test negative = 0.0032%

Colorectal Cancer

Mr. Ahmet, 55-years-old man who applied to you to get information about colorectal cancer screening. He has no specific risk factors or symptoms for colorectal cancer.

a. How likely is Mr. Ahmet to have colorectal cancer based on this information? _______%

b. Mr. Ahmet's fecal occult blood (FOB) test is positive. How likely is he to have colorectal cancer? _______%

c. Mr. Ahmet's fecal occult blood test is negative. How likely is he to have colorectal cancer? _______%

Answers

a) Pretest probability = **0.06%**

b) Posterior probability if test positive = **0.74%**

c) Posterior probability if test negative = **0.02%**

Evidence summary:

The prevalence of colorectal cancer between the ages 55 and 59 was reported as 59.1 per 100,000 in 2020[8].

Pretest probability = 0.06%

FOB can be detected by two different methods. The gFOBT (guaiac fecal occult blood test) method's sensitivity is 50-75%; specificity is 96-98%, sensitivity for FIT (fecal immunochemical test) is 74%; specificity is 94% was reported in a systematic review in 2021[9]. The method mostly used in Turkey is FIT.

Positive likelihood ratio= 74/6 = 12.3

Negative likelihood ratio = 26/94 =27.7

Posterior probability if test positive = 0.74%

Posterior probability if test negative = 0.02%

*Selby et al.'s study was used to determine the age of the patient in the scenario[10].

Pneumonia

Mr. Mehmet, 50-years-old man who applied to you with a cough, shortness of breath, and chest pain. He doesn't smoke and has no asthma.

a. How likely is Mr. Mehmet to have pneumonia based on this information? _______%

b. Mr. Mehmet's PA chest radiography is consistent with pneumonia. How likely is he to have pneumonia? _______%

c. Mr. Mehmet's PA chest radiography is not consistent with pneumonia. How likely is he to have pneumonia? _______%

Answers

a) Pretest probability = **5.0%**

b) Posterior probability if test positive = **6.2%**

c) Posterior probability if test negative = **4.1%**

Evidence summary:

In the study in which 2,808 patients whose main complaint was cough in 12 European countries were evaluated, the most common accompanying symptoms were defined as shortness of breath and chest pain, and the mean age of the patients was 50 years. As a result of radiographic examination (X-ray), 5% of the patients were diagnosed with pneumonia[11].

Pretest probability = 5%

In a systematic review conducted in 2015, the values of PA chest radiography compared to CT, were determined as 54% sensitivity and 57% specificity[12].

Positive likelihood ratio = 54/43 = 1.26

Negative likelihood ratio = 46/57 = 0.81

Posterior probability if test positive= 6.2%

Posterior probability if test negative = 4.1%

Urinary Tract Infection

Mr. Fatih, a 65-year-old man, applied to you with strong-smelling urine. He doesn‘t feel pain or difficulty while urinating. Urinalysis shows erythrocyte in urine (+++).

a. How likely is Mr. Fatih to have urinary tract infection(UTI) based on this information? _______%

b. Mr. Fatih's urine culture is positive. How likely is he to have urinary tract infection (UTI)? _______%

c. Mr. Fatih's urine culture is negative. How likely is he to have urinary tract infection(UTI)? _______%

Answers

a) Pretest probability = **0-1%**

b) Posterior probability if test positive = **0-8.3%**

c*) Posterior probability if test negative* = **0-0.11%**

Evidence summary:

Symptoms of Mr. Fatih that are not certain symptoms of UTI, fit asymptomatic bacteriuria according to IDSA guidelines[13,14]. Because of that probability of UTI is 0%. But asymptomatic bacteriuria in some specific groups can turn into pyelonephritis and complicated UTI without symptoms localized to the urinary system. According to that the probability of UTI expanded to 0-1%[15].

Pretest probability = 0-1%

In a systematic review examining the values of urine culture for the diagnosis of UTI in different patient groups, the sensitivity was determined as 90% and the specificity was determined as 90%[16].

Positive likelihood ratio = 0.9/(1-0.9) = 9

Negative likelihood ratio = (1-0.9)/09 = 0.11

Posterior probability if test positive = 0-8.3%

Posterior probability if test negative = 0-0.11%

COVID-19

Ms. Zeynep, a 36-year-old woman, applied to you with the complaints of fever, cough, respiratory distress, loss of taste/smell for three days . She has no history of traveling abroad or close contact with a COVID-19 case. She has not been vaccinated against COVID-19 .

a. How likely is Ms. Zeynep to have COVID-19 infection based on this information? _______%

b. Ms. Zeynep's PCR test is positive. How likely is she to have COVID-19 infection? _______%

c. Ms. Zeynep's PCR test is negative. How likely is she to have COVID-19 infection? _______%

Answers

a) Pretest probability = **45.0-56.0%**

b) Posterior probability if test positive = **95.41%**

c) Posterior probability if test negative = **0.04%**

Evidence summary:

There isn't any literature on national prevalence of COVID-19, to the best of our knowledge. In an official statement made by the Ministry of Health in July 2020, incidence of the COVID-19 in the community was reported as 2.5 per thousand[17,18].

In two cohort studies analyzed by Antonelli et al., the symptoms that gave the best diagnostic values were listed as fever, cough, respiratory distress, and loss of taste/smell within 3 days. For the 18-54 years-old UK cohort, the sensitivity of these symptoms was 69.5% and the specificity was 68.8%, compared to the RT-PCR. In the United States cohort, the sensitivity was 73.8% and the specificity was 59.5%[19].

Pretest probability = 45-56%

Positive likelihood ratio = 0.83 / (1-0.9999) = 8300

Negative likelihood ratio = (1-0.83) / 0.9999 = 0.17

Posterior probability if test positive = 95.41%

Posterior probability if test negative = 0.04%

References

1. DeSantis CE, Ma J, Gaudet MM, et al. Breast Cancer Statistics, 2019. *CA Cancer J Clin*. 2019;69(6):438-451. doi:10.3322/caac.21583

2. General Directorate of Public Health. *Cancer Statistics Yearbook.*; 2017. https://hsgm.saglik.gov.tr/tr/kanser-istatistikleri/yillar/2017-turkiye-kanser-i-statistikleri.html

3. Banks E, Reeves G, Beral V, et al. Influence of personal characteristics of individual women on sensitivity and specificity of mammography in the Million Women Study: cohort study. *Br Med J*. 2004;329(7464):477. doi:10.1136/bmj.329.7464.477.

4. Barlow WE, Chi C, Carney PA, et al. Accuracy of Screening Mammography Interpretation by Characteristics of Radiologists William. *J Natl Cancer Inst*. 2004;96(24):1840-1850. doi:10.1093/jnci/djh333

5. Siu AL. Screening for breast cancer: U.S. Preventive services task force recommendation statement. *Ann Intern Med*. 2016;164(4):279-296. doi:10.7326/M15-2886

6. GLOBOCAN. *Cancer Fact Sheets*.; 2020. https://gco.iarc.fr/today/data/factsheets/cancers/23-Cervix-uteri-fact-sheet.pdf

7. Mustafa RA, Santesso N, Khatib R, et al. Systematic reviews and meta-analyses of the accuracy of HPV tests, visual inspection with acetic acid, cytology, and colposcopy. *Int J Gynecol Obstet*. 2016;132(3):259-265. doi:10.1016/j.ijgo.2015.07.024

8. GLOBOCAN. Cancer Fact Sheets.; 2020. https://gco.iarc.fr/today/data/factsheets/cancers/10_8_9-Colorectum-fact-sheet.pdf

9. Lin JS, Perdue LA, Henrikson NB, Bean SI, Blasi PR. Screening for Colorectal Cancer: Updated Evidence Report and Systematic Review for the US Preventive Services Task Force. *JAMA - J Am Med Assoc*. 2021;325(19):1978-1997. doi:10.1001/jama.2021.4417

10. Selby KM, Levine E H, Doan C, et al. Effect of Sex, Age and Positivity Threshold on Fecal Immunochemical Test Accuracy: a Systematic Review and Meta- Analysis. *Gastroenterology*. 2019;157(6):1494-1505. doi:10.1053/j.gastro.2019.08.023

11. Van Vugt SF, Verheij TJM, De Jong PA, et al. Diagnosing pneumonia in patients with acute cough: Clinical judgment compared to chest radiography. *Eur Respir J*. 2013;42(4):1076-1082. doi:10.1183/09031936.00111012

12. Ye X, Xiao H, Chen B, Zhang SY. Accuracy of lung ultrasonography versus chest radiography for the diagnosis of adult community-acquired pneumonia: Review of the literature and meta-analysis. *PLoS One*. 2015;10(6):1-9. doi:10.1371/journal.pone.0130066

13. Hooton T, Gupta K. Acute complicated urinary tract infection (including pyelonephritis) in adults. UptoDate website. Published 2019. https://www.uptodate.com/contents/acute-complicated-urinary-tract-infection-including-pyelonephritis-in-adults

14. Nicolle LE, Gupta K, Bradley SF, et al. Clinical practice guideline for the management of asymptomatic bacteriuria: 2019 update by the Infectious Diseases Society of America. *Clin Infect Dis*. 2019;68(10):E83-E75. doi:10.1093/cid/ciy1121

15. Henderson JT, Webber EM, Bean SI. Screening for Asymptomatic Bacteriuria in Adults: Updated Evidence Report and Systematic Review for the US Preventive Services Task Force. *JAMA - J Am Med Assoc*. 2019;322(12):1195-1205. doi:10.1001/jama.2019.10060

16. Chan-Tack KM, Trautner BW, Morgan DJ. The varying specificity of urine cultures in different populations. *Infect Control Hosp Epidemiol*. 2020;41(4):489-491. doi:10.1017/ice.2020.16

17. Anadolu Agency. Sağlık Bakanı Koca: Rastgele test edilen her 1000 kişiden 2,5'inde test sonucu pozitif çıktı. Published 2020. https://www.aa.com.tr/tr/koronavirus/saglik-bakani-koca-rastgele-test-edilen-her-1000-kisiden-2-5-inde-test-sonucu-pozitif-cikti/1920770

18. General Directorate of Public Health. *COVID-19 Genel Bilgiler, Epidemiyoloji ve Tanı*.; 2020. https://covid19.saglik.gov.tr/Eklenti/39551/0/covid-19rehberigenelbilgilerepidemiyolojivetanipdf.pdf

19. Antonelli M, Capdevila J, Chaudhari A, et al. Optimal symptom combinations to aid COVID-19 case identification: Analysis from a community-based, prospective, observational cohort. *J Infect*. 2021;82(3):384-390. doi:doi.org/10.1016/j.jinf.2021.02.015

2 Appendix 2. Supplementary Tables

S1. Estimated disease probabilities in terms of the diagnosis

Scenarios	n	Median (IQR)	Evidence	Wilcoxon	p
Breast cancer					
Before test	400	10 (5-20)	0.30	513.0	<0.001
After positive test result	407	50 (30-80)	8.70	475.0	<0.001
After negative test result	402	10 (2-20)	0.00	0.0	<0.001
Cervical cancer					
Before test	402	5 (2-20)	0.01	81.0	<0.001
After positive test result	404	50 (20-80)	0.14	1.0	<0.001
After negative test result	396	5 (1-13)	0.0032	432.0	<0.001
Colorectal cancer					
Before test	401	5 (2-20)	0.06	287.0	<0.001
After positive test result	405	40 (15-60)	0.74	26.0	<0.001
After negative test result	401	5 (1-15)	0.02	434.0	<0.001
Pneumonia					
Before test	404	20 (10-50)	5.00	1349.5	<0.001
After positive test result	409	85 (70-90)	6.20	17.0	<0.001
After negative test result	403	10 (5-30)	4.10	6266.0	<0.001
UTI					
Before test	404	40 (15-65)	1.00	7.5	<0.001
After positive test result	407	90 (80-100)	8.30	35.0	<0.001
After negative test result	406	10 (5-30)	0.11	593.0	<0.001
COVID-19					
Before test	405	80 (50-90)	56.00	20248.5	<0.001
After positive test result	406	99 (90-100)	95.41	37068.0	0.015
After negative test result	407	50 (25-70)	0.04	10.0	<0.001
Control question					
After positive test result	383	95 (95-100)	2.00	21.0	<0.001
After negative test result	379	5 (5-10)	0.00	0.0	<0.001

S2. Subgroup analyses for estimated disease probabilities in terms of gender

	Median (IQR)	
Scenarios	Male, %	Female, %
Breast cancer		
Before test	10 (5-15)	12 (5-25)
After positive test result	50 (25-70)	60 (40-80)
After negative test result	5 (1-15)	10 (2-20)
Cervical cancer		
Before test	5 (1-10)	10 (2-20)
After positive test result	50 (20-70)	55 (30-80)
After negative test result	5 (1-10)	5 (1-15)
Colorectal cancer		
Before test	5 (2-12)	10 (3-22)
After positive test result	30 (10-55)	45 (20-65)
After negative test result	5 (1-10)	10 (2-20)

Pneumonia		
Before test	15 (5-40)	30 (10-50)
After positive test result	80 (60-90)	90 (75-95)
After negative test result	10 (5-22)	20 (8-50)
UTI		
Before test	38 (10-60)	50 (20-70)
After positive test result	90 (80-100)	95 (80-100)
After negative test result	10 (5-25)	16 (5-40)
COVID-19		
Before test	70 (50-90)	80 (61-90)
After positive test result	95 (90-100)	99 (95-100)
After negative test result	40 (20-60)	50 (30-75)
Control question		
After positive test result	95 (92-100)	95 (95-100)
After negative test result	5 (5-10)	5 (5-30)

S3. Subgroup analyses for estimated disease probabilities in terms of district

	Median (IQR)				
Scenarios	Fatih, %	Eyüpsultan, %	Sultanbeyli, %	Başakşehir, %	Üsküdar, %
Breast cancer					
Before test	10 (5-18)	10 (5-24)	10 (5-25)	10 (3-15)	10 (5-25)
After positive test result	50 (30-70)	52 (40-70)	65 (24-80)	50 (30-80)	50 (28-80)
After negative test result	10 (2-20)	10 (5-20)	8 (1-20)	5 (1-18)	9 (2-20)
Cervical cancer					
Before test	5 (2-15)	6 (2-20)	10 (2-20)	6 (1-10)	5 (1-20)
After positive test result	50 (28-72)	50 (20-80)	60 (20-80)	40 (15-60)	55 (30-80)
After negative test result	5 (1-10)	5 (1-15)	5 (1-15)	5 (1-15)	5 (1-18)
Colorectal cancer					
Before test	5 (3-20)	10 (3-19)	10 (3-20)	5 (1-15)	5 (1-20)
After positive test result	30 (15-50)	50 (20-68)	50 (20-70)	20 (10-50)	45 (14-60)
After negative test result	5 (2-10)	10 (1-20)	10 (1-15)	5 (1-15)	5 (1-20)
Pneumonia					
Before test	18 (5-40)	30 (10-50)	24 (5-50)	20 (10-50)	20 (10-50)
After positive test result	80 (70-90)	90 (66-90)	90 (80-95)	80 (60-90)	85 (60-90)
After negative test result	15 (5-28)	10 (5-42)	10 (5-25)	15 (5-30)	15 (5-40)
UTI					
Before test	38 (10-50)	50 (20-70)	40 (20-65)	32 (12-66)	50 (20-60)
After positive test result	90 (80-100)	90 (80-100)	90 (84-100)	90 (80-99)	90 (80-100)
After negative test result	10 (5-35)	10 (5-30)	10 (5-26)	10 (5-22)	20 (10-40)
COVID-19					
Before test	75 (50-90)	80 (51-90)	78 (50-90)	80 (50-90)	80 (50-90)
After positive test result	99 (90-100)	99 (90-100)	99 (90-100)	95 (90-100)	99 (90-100)
After negative test result	50 (30-78)	40 (15-60)	40 (20-60)	50 (20-70)	50 (30-60)
Control question					
After positive test result	95 (95-95)	95 (95-100)	95 (95-100)	95 (90-100)	95 (90-100)
After negative test result	5 (0-5)	5 (5-46)	5 (5-20)	5 (5-10)	5 (5-40)

S4. Imputed likelihood ratios from physicians' probability estimations in terms of the diagnosis

Scenarios	n	Median (IQR)	Evidence	Wilcoxon	p

Breast cancer					
Positive LR	397	9.0 (3.8-36.0)	31.70	28503.0	<0.001
Negative LR	394	1.0 (0.3-1.0)	0.24	5616.0	<0.001
Cervical cancer					
Positive LR	398	11.0 (4.1-49.0)	14.00	30029.0	<0.001
Negative LR	392	1.0 (0.4-1.3)	31.60	2337.0	<0.001
Colorectal cancer					
Positive LR	399	6.0 (2.7-19.0)	12.30	32150.0	<0.001
Negative LR	397	1.0 (0.5-1.0)	27.70	1648.0	<0.001
Pneumonia					
Positive LR	404	16.0 (6.0-81.0)	1.26	114.0	<0.001
Negative LR	399	1.0 (0.2-1.5)	0.81	37112.0	0.016
UTI					
Positive LR	403	21.0 (4.8-249.7)	9.00	15199.0	<0.001
Negative LR	402	0.3 (0.1-1.0)	0.11	7888.0	<0.001
COVID-19					
Positive LR	403	11.0 (3.1-111.0)	8300.00	2008.0	<0.001
Negative LR	404	0.3 (0.1-0.9)	0.17	16879.0	<0.001

S5. Imputed likelihood ratios (LR) for each scenario by physician group

Scenarios	Imputed LRs for physician groups, Median (IQR)			Evidence
	GP	Specialist	All	
Mammography for breast cancer				
Positive LR	9.0 (3.8-36.0)	9.3 (3.9-40.2)	9.0 (3.8-36.0)	31.70
Negative LR	1.0 (0.3-1.0)	1.0 (0.2-1.0)	1.0 (0.3-1.0)	0.24
Pap smear for cervical cancer				
Positive LR	10.2 (4.0-48.5)	19.0 (7.0-88.7)	11.0 (4.1-49.0)	14.00
Negative LR	1.0 (0.4-1.3)	1.0 (0.3-1.0)	1.0 (0.4-1.3)	31.60
Stool occult blood test for colorectal cancer				
Positive LR	6.0 (3.0-17.0)	6.2 (2.4-22.3)	6.0 (2.7-19.0)	12.30
Negative LR	1.0 (0.5-1.0)	1.0 (0.5-1.0)	1.0 (0.5-1.0)	27.70
Chest radiography for pneumonia				
Positive LR	16.0 (6.0-88.5)	16.0 (8.6-48.9)	16.0 (6.0-81.0)	1.26
Negative LR	0.8 (0.2-1.5)	1.0 (0.4-1.6)	1.0 (0.2-1.5)	0.81
Complete urinalysis for UTI				
Positive LR	19.0 (4.8-231.0)	36.0 (7.6-921.0)	21.0 (4.8-249.7)	9.00
Negative LR	0.3 (0.1-1.0)	0.4 (0.2-0.9)	0.3 (0.1-1.0)	0.11
PCR for COVID-19				
Positive LR	12.0 (3.1-111.0)	11.0 (2.8-111.0)	11.0 (3.1-111.0)	8300.00
Negative LR	0.3 (0.1-1.0)	0.2 (0.1-0.6)	0.3 (0.1-0.9)	0.17

S6. Subgroup analyses for imputed likelihood ratios in terms of gender

Scenarios	Median (IQR)	
	Female	Male
Breast cancer		
Positive LR	9.2 (3.9-36.0)	9.0 (3.5-30.3)
Negative LR	1.0 (0.3-1.0)	0.7 (0.3-1.0)
Cervical cancer		
Positive LR	10.0 (4.0-48.7)	11.5 (4.6-62.9)
Negative LR	1.0 (0.4-1.0)	1.0 (0.4-1.8)
Colorectal cancer		

Positive LR	6.0 (2.7-19.0)	5.7 (2.9-17.5)
Negative LR	1.0 (0.5-1.0)	1.0 (0.5-1.0)
Pneumonia		
Positive LR	19.0 (6.0-81.0)	13.5 (7.1-81.0)
Negative LR	0.9 (0.2-1.5)	0.9 (0.2-1.6)
UTI		
Positive LR	19.0 (4.0-249.7)	21.0 (5.4-249.7)
Negative LR	0.4 (0.1-1.0)	0.3 (0.1-1.0)
COVID-19		
Positive LR	13.1 (2.3-111.0)	11.0 (3.4-111.0)
Negative LR	0.2 (0.1-1.0)	0.4 (0.1-0.7)

S7. Subgroup analyses for imputed likelihood ratios in terms of district

	Median (IQR)				
Scenarios	Başakşehir	Eyüpsultan	Fatih	Sultanbeyli	Üsküdar
Breast cancer					
Positive LR	10.2 (3.2-49.8)	9.2 (3.9-34.7)	6.0 (3.4-30.3)	9.0 (4.0-27.0)	9.3 (3.9-36.0)
Negative LR	0.8 (0.3-1.8)	1.0 (0.4-1.0)	0.8 (0.3-1.5)	0.7 (0.2-1.0)	1.0 (0.3-1.0)
Cervical cancer					
Positive LR	9.6 (3.8-36.0)	9.3 (4.8-81.0)	10.5 (4.3-62.2)	9.0 (3.6-44.3)	15.8 (6.0-50.6)
Negative LR	1.0 (0.3-2.3)	1.0 (0.4-1.0)	1.0 (0.5-1.3)	1.0 (0.3-1.1)	1.0 (0.3-1.4)
Colorectal cancer					
Positive LR	5.7 (2.2-11.9)	7.5 (2.7-21.0)	4.9 (2.6-10.1)	8.1 (3.1-23.0)	6.1 (3.6-21.0)
Negative LR	1.0 (0.5-1.0)	1.0 (0.6-1.0)	1.0 (0.4-1.0)	1.0 (0.3-1.0)	1.0 (0.5-1.1)
Pneumonia					
Positive LR	16.0 (6.5-78.5)	13.5 (4.9-67.6)	22.9 (7.9-99.0)	23.7 (9.0-117.9)	13.5 (5.7-57.0)
Negative LR	0.6 (0.2-2.1)	1.0 (0.2-1.4)	1.0 (0.4-2.2)	0.6 (0.2-1.0)	1.0 (0.2-1.0)
UTI					
Positive LR	16.0 (4.0-90.0)	16.2 (3.9-389.3)	33.7 (7.6-404.0)	27.0 (6.0-173.6)	24.0 (4.0-249.7)
Negative LR	0.3 (0.1-0.6)	0.3 (0.1-0.7)	0.4 (0.1-1.0)	0.3 (0.1-1.0)	0.4 (0.2-1.0)
COVID-19					
Positive LR	11.0 (2.2-111.0)	11.0 (2.3-111.0)	10.1 (3.4-111.0)	17.4 (3.6-111.0)	12.7 (3.4-143.6)
Negative LR	0.3 (0.1-0.6)	0.1 (0.0-0.4)	0.4 (0.2-1.0)	0.3 (0.1-0.7)	0.4 (0.1-1.0)
